# An *in vitro* assessment of panel of engineered nanomaterials using a human renal cell line: cytotoxicity, pro-inflammatory response, oxidative stress and genotoxicity

**DOI:** 10.1186/1471-2369-14-96

**Published:** 2013-04-25

**Authors:** Ali Kermanizadeh, Sandra Vranic, Sonja Boland, Kevin Moreau, Armelle Baeza-Squiban, Birgit K Gaiser, Livia A Andrzejczuk, Vicki Stone

**Affiliations:** 1 Heriot-Watt University, School of Life Sciences, John Muir building, Edinburgh, EH14 4AS, UK; 2 Laboratoire Biologie Fonctionnelle et Adaptative, Equipe Réponses Moléculaires et Cellulaires aux Xénobiotiques, Université Paris Diderot-Paris 7, 5 rue Thomas Mann, Paris cedex 13, 75 205, France

**Keywords:** Kidneys, Nanotoxicity, Inflammation, ROS, DNA damage

## Abstract

**Background**

It has been shown that nanomaterials (NMs) are able to translocate to secondary tissues one of the important being the kidneys. Oxidative stress has been implicated as a possible mechanism for NM toxicity, hence effects on the human renal proximal tubule epithelial cells (HK-2) treated with a panel of engineered nanomaterials (NMs) consisting of two zinc oxide particles (ZnO - coated - NM 110 and uncoated - NM 111), two multi walled carbon nanotubes (MWCNT) (NM 400 and NM 402), one silver (NM 300) and five TiO_2_ NMs (NM 101, NRCWE 001, 002, 003 and 004) were evaluated.

**Methods**

In order to assess the toxicological impact of the engineered NMs on HK-2 cells - WST-1 cytotoxicity assay, FACSArray, HE oxidation and the comet assays were utilised. For statistical analysis, the experimental values were compared to their corresponding controls using an ANOVA with Tukey’s multiple comparison.

**Results**

We found the two ZnO NMs (24 hr LC_50_ – 2.5 μg/cm^2^) and silver NM (24 hr LC_50_ – 10 μg/cm^2^) were highly cytotoxic to the cells. The LC_50_ was not attained in the presence of any of the other engineered nanomaterials (up to 80 μg/cm^2^). All nanomaterials significantly increased IL8 and IL6 production. Meanwhile no significant change in TNF-α or MCP-1 was detectable. The most notable increase in ROS was noted following treatment with the Ag and the two ZnO NMs. Finally, genotoxicity was measured at sub-lethal concentrations. We found a small but significant increase in DNA damage following exposure to seven of the ten NMs investigated (NM 111, NRCWE 001 and NRCWE 003 being the exception) with this increase being most visible following exposure to Ag and the positively charged TiO_2_.

**Conclusions**

While the NMs could be categorised as low and highly cytotoxic, sub-lethal effects such as cytokine production and genotoxicity were observed with some of the low toxicity materials.

## Background

The prefix “nano” was specifically coined for particles containing tens or hundreds of atoms, with dimensions at the scale of less than 100 nm [1]. It is this small size which is fundamental to the field of nanotechnology, although other physicochemical properties influence the physical, biological and toxicological properties of these manufactured materials [2]. The principal reason for producing and exploiting nanomaterials (NMs) is that their behaviour is fundamentally different from the bulk form of the same compound [2]. Accordingly, previously unexploited beneficial properties of a given material can be explored in its nano size range [2]. As a result the amount of interest in nanotechnology has risen exponentially across many fields aiming to exploit a wealth of opportunities. These applications include medicine, cosmetics, textiles, electronics and engineering. However, there is concern that the increased release and exposure to these engineered NMs could be potentially hazardous in an occupational and consumer setting [3-5]. A thorough risk evaluation is urgently required to provide an improved understanding of the implication of exposure to different NMs and any potential threat to humans or the environment [6]. A very effective strategy for achieving this is in the form of a critical risk assessment. Risks are assessed based upon the level of exposure to the manufactured NM, toxicology (hazard) of the material in question, route of exposure and its bio-persistence. Hence, it is necessary to identify the hazards associated with NM exposure both *in vitro* and *in vivo*, to assemble a knowledge base on the potential toxicity associated with NM exploitation on human health [4].

It is widely accepted that the skin, lungs and the gastrointestinal tract (GIT) are in constant contact with the external environment, and it is thus not surprising to find all three systems being primary exposure sites for NMs [5]. However following exposure some NMs are able to translocate to secondary target organs located at a distance from the original point of entry, which includes the kidneys [7,8].

The kidneys are principally responsible for the removal of metabolic waste such as urea and ammonia. However it is believed that other waste products and toxic substances such as NMs could also be excreted through urine [9]. The kidneys receive blood from the renal arteries which branch directly from the dorsal aorta. Despite their relatively small size, the kidneys receive approximately 20% of the entire cardiac output [10], making the organs highly susceptible to xenobiotics such as NMs [11]. In theory both the glomerular structures (during plasma filtration) and tubular epithelial cells may be exposed to NMs. Since the major function of the kidneys is to eliminate a variety of potentially harmful substances (including the potential excretion of NMs), these organs are extremely important targets for investigation with regards to nanomaterial exposure and hazard.

Previous experiments have demonstrated that mice exposed to copper NMs had severe damage to the proximal tubular cells [11], while Wang and colleagues observed glomerulonephritis and pathological degeneration of the renal proximal convoluted tubules following oral TiO_2_ administration [12]. Proximal tubule cells exhibit well developed basal infolding and an apical brush border, enabling intense pinocytic activity and variable transport and co-transport of particles [13], therefore these cells would be an important target for a nanotoxicological investigation. Hence, this *in vitro* study conducted as part of the European funded project ENPRA (Risk assessment of engineered nanoparticles) investigated the adverse effects of a panel of widely utilised engineered NMs (five TiO_2_, two ZnO, two MWCNTs and one Ag) on the immortalized adult human renal proximal tubule epithelial cells (HK-2) with respect to cytotoxicity, oxidative stress, cytokine secretion and DNA damage.

## Methods

### Nanomaterials

Nanomaterials were purchased as stated: NM 101 (Hombikat UV100; rutile with minor anatase; 7 nm), NM 110 (BASF Z-Cote; zincite, uncoated, 100 nm), NM 111 (BASF Z-Cote; zincite coated with triethoxycaprylylsilane, 130 nm), NM 300 (RAS GmbH; Ag capped with polyoxylaurat Tween 20 - < 20 nm), NM 400 (Nanocyl; entangled MWCNT, diameter 30 nm, length 5 μm), NM 402 (Arkema Graphistrength C100; entangled MWCNT, diameter 30 nm, length 20 μm). The above mentioned nanomaterials were sub-sampled under Good Laboratory Practice conditions and preserved under argon in the dark until use. These NMs were received from the European Commission Joint Research Centre (Ispra, Italy). The NRCWE samples were procured by the National Research Centre for the Working Environment. Sub-sampling was completed into 20 ml Scint-Burk glass pp-lock with Alu-Foil (WHEA986581; Wheaton Industries Inc.) after pooling and mixing of the material. NRCWE 001, TiO_2_ rutile 10 nm was purchased from NanoAmor (Houston, USA) and also used for production of NRCWE 002 (TiO_2_ rutile 10 nm with positive charge) and NRCWE 003 (TiO_2_ rutile 10 nm with negative charge) using the procedures described previously [14]. NRCWE 004 (TiO_2_ rutile 94 nm) was purchased from NaBond.

### Characterisation of the panel of engineered nanomaterials

All the nanomaterials used in this study were characterised by a combination of analytical techniques in order to infer primary physical and chemical properties useful to understand their toxicological behaviour. A comprehensive list of the main physical and chemical properties of the panel NMs has been shown (Table 1) [Reproduced and modified from 14]. Furthermore the hydrodynamic size distributions and zeta potential of the NMs dispersed in the complete renal cell medium (K-SFM) and RPMI with 10% FCS (RPMI-FCS) were determined in the 1–128 μg/ml concentration range by Dynamic Light Scattering (DLS) using a Malvern Metasizer nano series – Nano ZS (USA) (Table 1).

**Table 1:** **Main physicochemical characteristics of engineered nanomaterials investigated - reproduced from**[14]

**ENM code**	**ENM type**	**Phase**	**XRD Size [nm]**	**TEM Size**	**Primary characteristics by TEM analysis**	**Surface area (BET) [m**^**2**^**/g]**	**Known coating**	**Size in K-SFM (DLS) Ψ**	**Size in RPMI-FCS (DLS) Ψ**	**Zeta potential K-SFM**	**Zeta potential RPMI - FCS**
NM101	TiO_2_	Anatase^€^	9	4-8/50-100	Two structures found; type 1 show agglomerates in the 50–1500 nm range	322	none	221	358	−11.4	−17.7
NM110	ZnO	Zincite	70 to > 100	20-250/50-350	Mainly 2 euhedral morphologies: 1) aspect ratio close to 1 (20–250 nm range and few particles of approx. 400 nm) 2) ratio 2 to 7.5 (50–350 nm). Minor amounts of particles with irregular morphologies observed.	14	none	393	453.6	−11.5	−13
NM111	ZnO	Zincite	58-93	20-200/10-450	As NM110, but with different size distributions. 1) particles with aspect ratio close to 1 (~90% in the 20–200 nm range); 2) particles with aspect ratio 2 to 8.5 (~90% in the 10–450 nm ratio).	18	Trie-othoxy-capry-lsilane 130	332	362.4	−11.4	−12.6
NM300	Ag	Ag	7^$^ 14^£^ <18/15/> 100^#^	8-47 (av.: 17.5)	Mainly euhedral NM; minor fractions have either elongated (aspect ratio up to ~ 5) or sub-spherical morphology	NA	none	87	51.59	−10.2	−13.3
NM400	MWCNT	-	-	D: 5-35 L: 700-3000	Irregular entangled kinked and mostly bent MWCNT (10–20 walls). Some CNTs were capped and some cases multiple caps were found due to overgrowth. Fe/Co catalysts (6–9 nm, average 7.5 nm) were found inside the tubes.	298	none	*	*	*	*
NM402	MWCNT	-	-	D: 6-20 L: 700-4000	Entangled irregular, mostly bent MWCNT (6–14 walls). Some tubes were capped by unknown material. Some nano-onions (5–10 nm) and amorphous carbon structures mixed with Fe (5–20 nm). Residual catalyst was observed. Individual catalyst particles up to 150 nm were also detected.	225	none	*	*	*	*
NRCWE001	TiO_2_	Rutile^§^	10	80-400	Irregular euhedral particles detected by TEM	99	none	349	337.5	−11.6	−14.7
NRCWE002	TiO_2_	Rutile	10	80-400	Irregular euhedral particles detected by TEM	84	Positive charged	314	378.8	−13.2	−12.7
NRCWE003	TiO_2_	Rutile	10	80-400	Irregular euhedral particles detected by TEM	84	Negative charged	384	423.6	−15.3	−13.2
NRCWE004	TiO_2_	Rutile	App. 100	1-4/10-100/100-200/1000-2000	Five different particle types were identified: 1) irregular spheres, 1–4 nm (av. Diameter); 2) irregular euhedral particles, 10–100 nm (longest dimension); 3) fractal-like structures in long chains, 100–200 nm (longest dimension); 4) big irregular polyhedral particles, 1-2 μm (longest dimension); 5) large irregular particles with jagged boundaries, 1–2 μm (longest dimension).			396	482.6	−11.3	−12.4

^€^ 1 percent rutile found in one of two samples analyzed.

^$^ wet XRD in capillary tube.

^£^ dried samples.

^#^ sample with deposits.

^§^ ca. 6% anatase was observed in one of two samples analyzed.

* Not detectable by DLS due to the very large aspect ratio.

Ψ Intensity based size average in biological media after 15 mins.

Zincite –mineral form of ZnO.

PDI (polydispersity index) values were under 0.45 for all given DLS values.

Abbreviations:

D, Diameter.

DLS, Dynamic Light Scattering.

ENM, Engineered nanomaterial.

L, Length.

XRD, X ray diffraction.

### Cell culture and treatment with nanomaterials

The immortalized adult human renal proximal tubule epithelial cells (HK-2) were obtained from the American Type Culture Collection (ATCC, USA). The cells were maintained in Keratinocyte Serum Free Medium (Gibco, UK) containing 25 μl of bovine (pituitary) extract, 0.2 ng/ml human recombinant epidermal growth factor and 100 U/ml penicillin/streptomycin (termed K-SFM) or RPMI (Sigma, France) with 10% FCS and 100 U/ml penicillin/streptomycin (termed RPMI-FCS), at 37°C and 5% CO_2_. Two different media with varying amounts of protein (K-SFM less than 2% proteins, RPMI-FCS 10% protein) were utilised to allow comparisons of the *in vitro* cytotoxicity to HK-2 cells.

The Ag was supplied in de-ionised water (85%) with 7% stabilizing agent (ammonium nitrate) and 8% emulsifiers (4% each of Polyoxyethylene Glycerol Trioleate and Tween 20). All other materials were supplied as dry powders. NMs were dispersed in MilliQ deionised water with 2% FCS. For the coated ZnO, the particles were wetted with 0.5% vol ethanol before the addition of the dispersion media. The nanomaterials were sonicated for 16 mins without pause following the protocol developed for ENPRA [15]. Following the sonication step, all samples were immediately transferred to ice.

To ascertain the toxicity of NMs to the HK-2 cells, ten concentrations between 0.16 and 80 μg/cm^2^ were used (corresponding to 0.5 to 256 μg/ml).

### WST-1 cell viability assay

The renal cell lines were seeded in 96 well plates 10^4^ cells per well in 100 μl of the cell culture medium and incubated for 24 hr in K-SFM or 48 hr with comp RPMI-FCS at 37°C and 5% CO_2_. The following day the cells were exposed to the materials or controls for 24 hr at 37°C, 5% CO_2_. Subsequent to NM treatment, cell supernatants were collected and frozen at −80°C and later used for FACS array analysis. Plates were washed twice with phosphate buffered saline (PBS), followed by the addition of 10 μl of the WST-1 cell proliferation reagent and 90 μl of fresh medium. Plates were then incubated for 1 hr at 37°C, 5% CO_2_. The supernatant was transferred to a fresh plate and the absorbance measured by dual wavelength spectrophotometry at 450 nm and 630 nm using a micro-plate reader.

### Production of IL6, IL8, TNF-α and MCP-1

The levels of human interleukin 6 (IL 6), IL8, tumour necrosis factor α (TNF-α) and monocyte chemo-attractant protein 1 (MCP-1) from HK-2 supernatants was measured using the BD^TM^ Cytometric Bead Array cytokine Flex sets (bead based immunoassay) according to the manufacturer’s instructions. A BD^TM^ FACSArray, USA was utilised for cytokine measurement.

### HE oxidation

The renal cells were seeded in 12 well plates (10^4^ cells per cm^2^ in 1 ml of medium) and incubated for 48 hr at 37°C and 5% CO_2_. Cells were exposed to the materials or controls for 4 hr at 37°C, 5% CO_2_. The treatment was removed and the cells were washed with complete medium. Cells were then harvested with 0.05% trypsin/EDTA and centrifuged at 300 g for 5 min before being re-suspended in phenol red free media containing 1 μM of dihydroethidium (DHE) (specific for superoxide production) (Sigma, France) for 30 min at 37°C in the dark and analysed by CyAn ADP LX DakoCytomation cytometer (Beckman Coulter, France). Excitation and emission wavelengths were 488 and 620 nm respectively. Minimum of 10000 cells was analyzed after exclusion of cellular debris and NMs from the analysis by gating on the 620 nm Log versus FS area graph.

### Detection of DNA strand breaks in HK-2 cells

The FPG (formamidopyrimidine [fapy] – DNA glycosylase) modified Comet assay was used to measure DNA strand breaks and specific oxidative DNA damage such as 7, 8-dihydro-8-oxoguanine, 8-oxoadenine, fapy-guanine etc., as previously described [16]. In this study the tail moment [17] was measured using an automatic image analyser (Comet Assay IV; Perceptive Instruments, UK) connected to a fluorescence microscope. Images were captured using a stingray (F-033B/C) black and white video camera.

Briefly, after a 4 hr NM treatment (or positive control - 60 μM of H_2_O_2_), the HK-2 cells were rinsed twice with PBS and detached using trypsin before being suspended in 5 ml of culture medium. Cells were centrifuged for 10 mins at 250 g, 4°C and re-suspended at a concentration of 1.5 × 10^6^ cells/ml in complete medium. A 20 μl volume of calculated cell suspension was added to 240 μl of 0.5% low melting point agarose. Next, 125 μl of the mixture was added to pre-coated slides (1.5% agarose) in triplicate. Following 10 mins of solidification on ice, slides were lyzed overnight at 4°C in lysis buffer (2.5 M NaCl, 100 mM EDTA, 10 mM Tris-base, pH 10, containing 10% DMSO and 1% TritonX-100). The slides were washed three times for 5 mins with FPG enzyme buffer (40 mM HEPES, 100 mM KCl, 0.5 mM EDTA, 0.2 mg/ml BSA - pH 8), covered with 100 μl of either buffer or FPG in buffer (1:30), sealed with a cover slip and incubated for 30 mins at 37°C. FPG cleaves DNA at locations of oxidation leading to a greater tail for cells exhibiting oxidative DNA damage [18]. All slides were then transferred into a black chilled electrophoresis tank. After alkaline unwinding (pH 13) for 20 mins, electrophoresis was performed for 15 mins at 270 mA, 24 V. Slides were neutralized three times for 5 mins using a neutralization buffer (0.4 M TrisBase, pH 7.5). Before analysis, slides were dried for 10 mins and stained with GelRed (2 in 10000, 40 μl per slide). A total of 50 cells were analyzed per slide per experiment.

### Statistical analysis

All data are expressed as mean ± standard error of the mean. For statistical analysis, the experimental results were compared to their corresponding control values using an ANOVA with Tukey’s multiple comparison. All statistical analysis was carried out utilizing Minitab 15. A p value of < 0.05 was considered to be significant. All experiments were repeated a minimum of three times.

## Results

### Characteristics of nanomaterials and exposure media

Investigated nanomaterials were characterised by a combination of analytical techniques in order to infer primary physical and chemical properties useful to understand their toxicological behaviour. A list of the measured physical and chemical properties is described (Table 1). In order to investigate if the nanomaterials behaved differently in K-SFM or RPMI-FCS, the hydrodynamic size distributions and zeta potential of the NMs dispersed in the two media was measured between a 1–128 μg/ml concentration range by DLS (Table 1). It is widely accepted that DLS is not a suitable method of ascertaining the size of carbon nanotubes (due to the fact that the machine measures size based on the principle that particles are spherical and well dispersed), hence we examined how the two MWCNTs behave in our two chosen media utilising light microscopy. We found that the MWCNTs agglomerated into larger clusters in the complete RPMI which contained higher levels of FCS in comparison to the K-SFM (data not shown). Our zeta potential data seems to demonstrate that these NMs have the tendency to aggregate in the media utilised in this study (Table 1).

### Impact of the selected panel of NMs on HK-2 cell viability

Our toxicity data show a dose dependent decrease in cell viability at 24 hr across the entire nanomaterial panel (Table 2). The Ag and the two ZnO NMs were shown to be highly cytotoxic (NM 111 LC_50_ 2.5 μg/cm^2^ – K-SFM, LC_50_ 0.9 μg/cm^2^ – RPMI-FCS; NM 110 LC_50_ 2.5 μg/cm^2^ – K-SFM, LC_50_ 1 μg/cm^2^ – RPMI-FCS; NM 300 10 μg/cm^2^ – K-SFM, LC_50_ 4.5 μg/cm^2^ – RPMI-FCS) after a 24 hr exposure. All of the TiO_2_ and MWCNT NMs were considered to be low toxicity materials as the LC_50_ was not reached after a 24 hr exposure to the HK-2 cells at the range investigated. We observed slightly higher cytotoxicity to the cells in RPMI-FCS exposed to the Ag and ZnO NMs. We also investigated the toxicity of the ENPRA dispersants namely NM 300 dispersant termed (NM 300 DIS) and 0.5% ethanol in complete medium. We found no cytotoxicity of any of these dispersants to the HK-2 cells (data not shown) so we conclude that all observed toxicity is due to exposure to the NMs investigated. LC_20_ are shown as these values were utilised for the FPG modified comet assay (Table 2).

**Table 2:** Cytotoxicity following exposure of the HK-2 cells in two different media to a panel of engineered nanomaterials

**NM**	**K-SFM**		**RPMI-FCS**	
	**LC**_**20**_	**LC**_**50**_	**LC**_**20**_	**LC**_**50**_
NM 111	0.8 μg/cm^2^	2.5 μg/cm^2^	0.5 μg/cm^2^	0.9 μg/cm^2^
NM 110	0.64 μg/cm^2^	2.5 μg/cm^2^	0.64 μg/cm^2^	1 μg/cm^2^
NM 300	1.25 μg/cm^2^	10 μg/cm^2^	1.25 μg/cm^2^	4.5 μg/cm^2^
NM 400	2.5 μg/cm^2^	Not reached up to 80 μg/cm^2^	20 μg/cm^2^	Not reached up to 80 μg/cm^2^
NM 402	4.5 μg/cm^2^	Not reached up to 80 μg/cm^2^	20 μg/cm^2^	Not reached up to 80 μg/cm^2^
NRCWE 002	20 μg/cm^2^	Not reached up to 80 μg/cm^2^	Not reached up to 80 μg/cm^2^	Not reached up to 80 μg/cm^2^
NRCWE 001	40 μg/cm^2^	Not reached up to 80 μg/cm^2^	Not reached up to 80 μg/cm^2^	Not reached up to 80 μg/cm^2^
NRCWE 004	40 μg/cm^2^	Not reached up to 80 μg/cm^2^	Not reached up to 80 μg/cm^2^	Not reached up to 80 μg/cm^2^
NRCWE 003	60 μg/cm^2^	Not reached up to 80 μg/cm^2^	Not reached up to 80 μg/cm^2^	Not reached up to 80 μg/cm^2^
NM 101	Not reached up to 80 μg/cm^2^	Not reached up to 80 μg/cm^2^	Not reached up to 80 μg/cm^2^	80 μg/cm^2^

The cells were exposed to the NM for 24 hr with cytotoxicity measured via WST-1 (NMs ranked in order of cytotoxicity to HK-2 cells – high to low).

### Cytokine secretion by HK-2 cells following exposure to the panel of NMs

**Figure 1:**
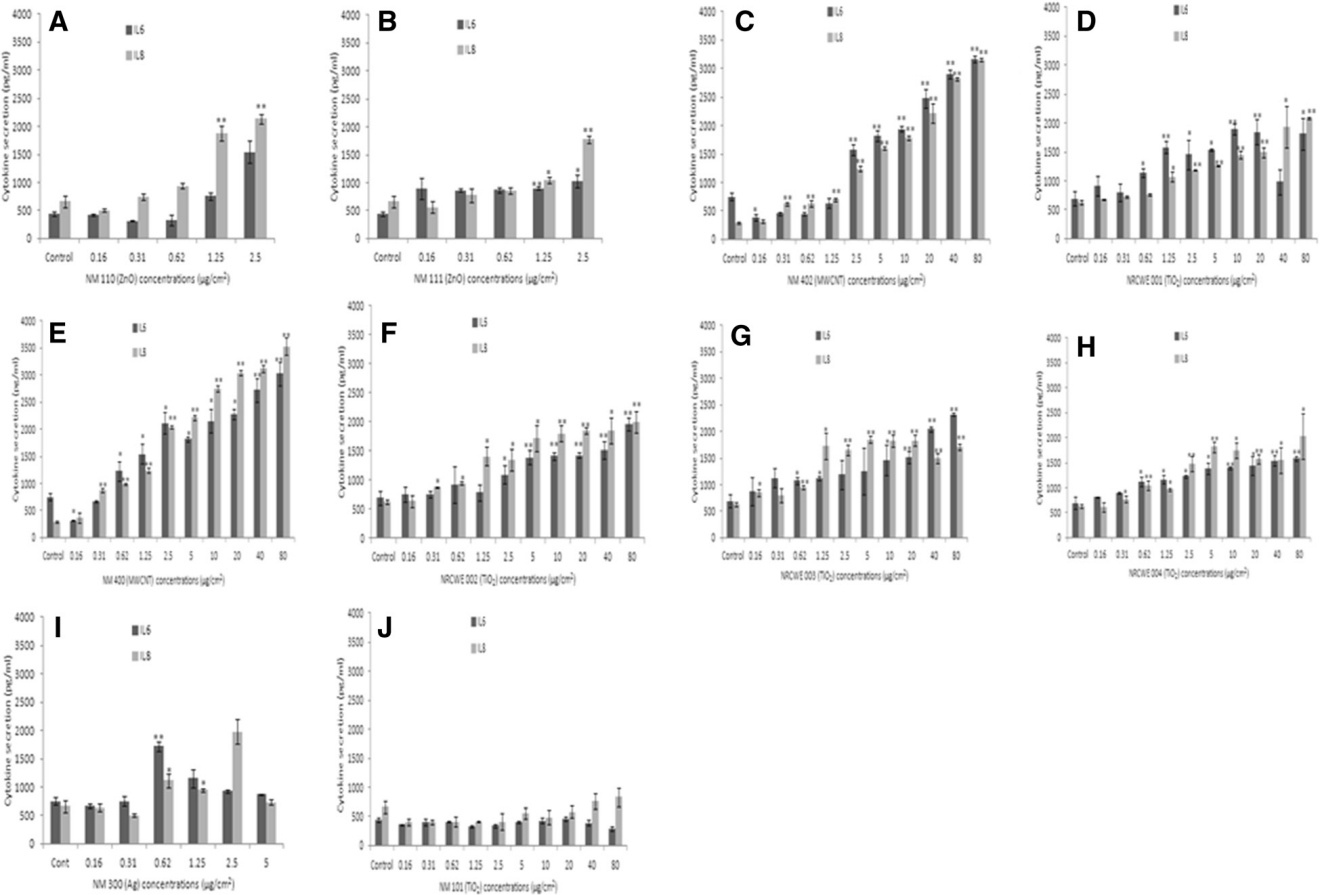
**IL6 (black bars) and IL8 (grey bars) production by HK-2 cells in the presence of the panel of engineered nanomaterials.** The cells were exposed to the sub-lethal concentrations of the NMs for 24 hr with cytokine secretion measured utilising the FACSArray. Values represent mean ± SEM (n = 3), significance indicated by * = p < 0.05 and ** = p < 0.005, when material treatments are compared to the control. **A**) NM 110 **B**) NM 111 **C**) NRCWE 402 **D**) NM 400 **E**) NRCWE 002 **F**) NRCWE 003 **G**) NRCWE 001 **H**) NRCWE 004 **I**) NM 300 **J**) NM 101. The Figure has been arranged according to the biological effect of individual NMs.

Changes in cytokine production as a consequence of NM exposure were assessed within the supernatant of exposed renal cells and quantified via FACSArray. We found a dose dependent (exposure to sub-lethal concentrations of the NMs) increase in the levels of IL8 and IL6 from the kidney cells with statistical significance being reached at the higher concentrations following exposure to nine of the ten nanomaterials (TiO_2_ - NM 101 being the exception) (Figure 1J). Additionally we found no change in the levels of MCP-1 or TNF-α secreted from the HK-2 cells following the 24 hr exposure to any of the selected NMs (data not shown).

### HE Oxidation

**Figure 2:**
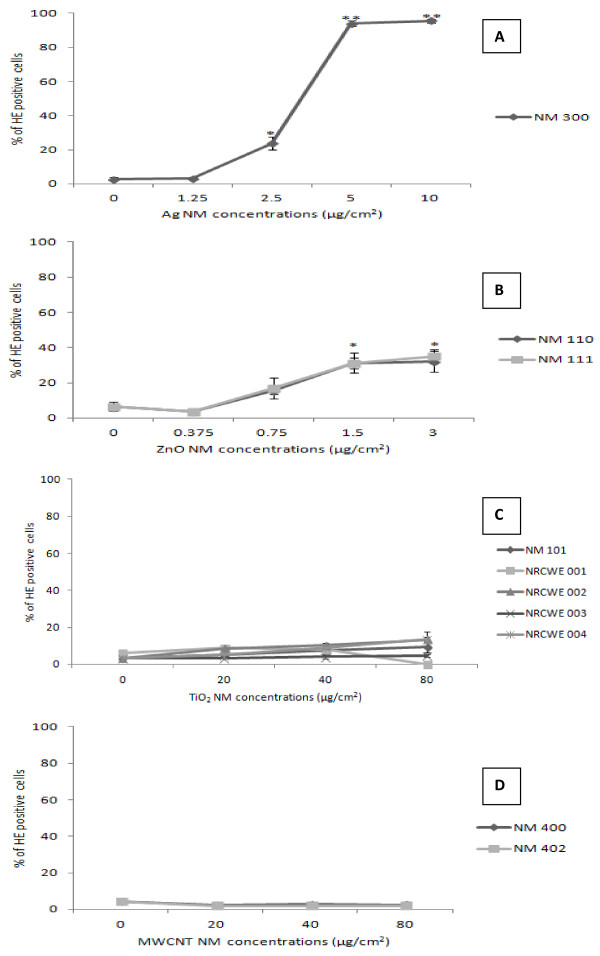
**Intracellular ROS production following exposure of the HK-2 cells to the panel of engineered nanomaterials.** The cells were exposed to the NM for 4 hr with oxidative stress measured via HE oxidation by flow cytometry. Values represent mean ± SEM (n = 6) **A**) Ag **-** NM 300 **B)** ZnO - NM 110 and NM 111 **C**) TiO_2_ - NM 101, NRCWE 001, 002, 003 and 004 **D**) MWCNT - NM 400 and NM 402.

Hydroethidium is the chemically reduced form of the commonly used DNA intercalating dye ethidium bromide [19]. This reduced dye is therefore useful for detection of oxidative activities in viable intact cells. Only once it is internalized and dehydrogenated (oxidized) to ethidium, it can intercalate into DNA. Normally due to their compromised membranes, only dead cells are typically labelled by ethidium bromide when it selectively binds to DNA. However HE is a neutral probe and is able to penetrate the cell membrane of live cells, staining their cytoplasm blue as well as the chromatin/nucleus of living cells red. We noted a significant increase in HE positive cells following exposure to Ag and ZnO NMs (Figure 2A and B). A very small yet significant increase was also observed to all the TiO_2_ NM with the exception of NRCWE 003 (Figure 2C). However no change in intracellular reactive oxygen species was noted following exposure to either of the MWCNTs (Figure 2D).

### DNA damage in the HK-2 cells

**Figure 3:**
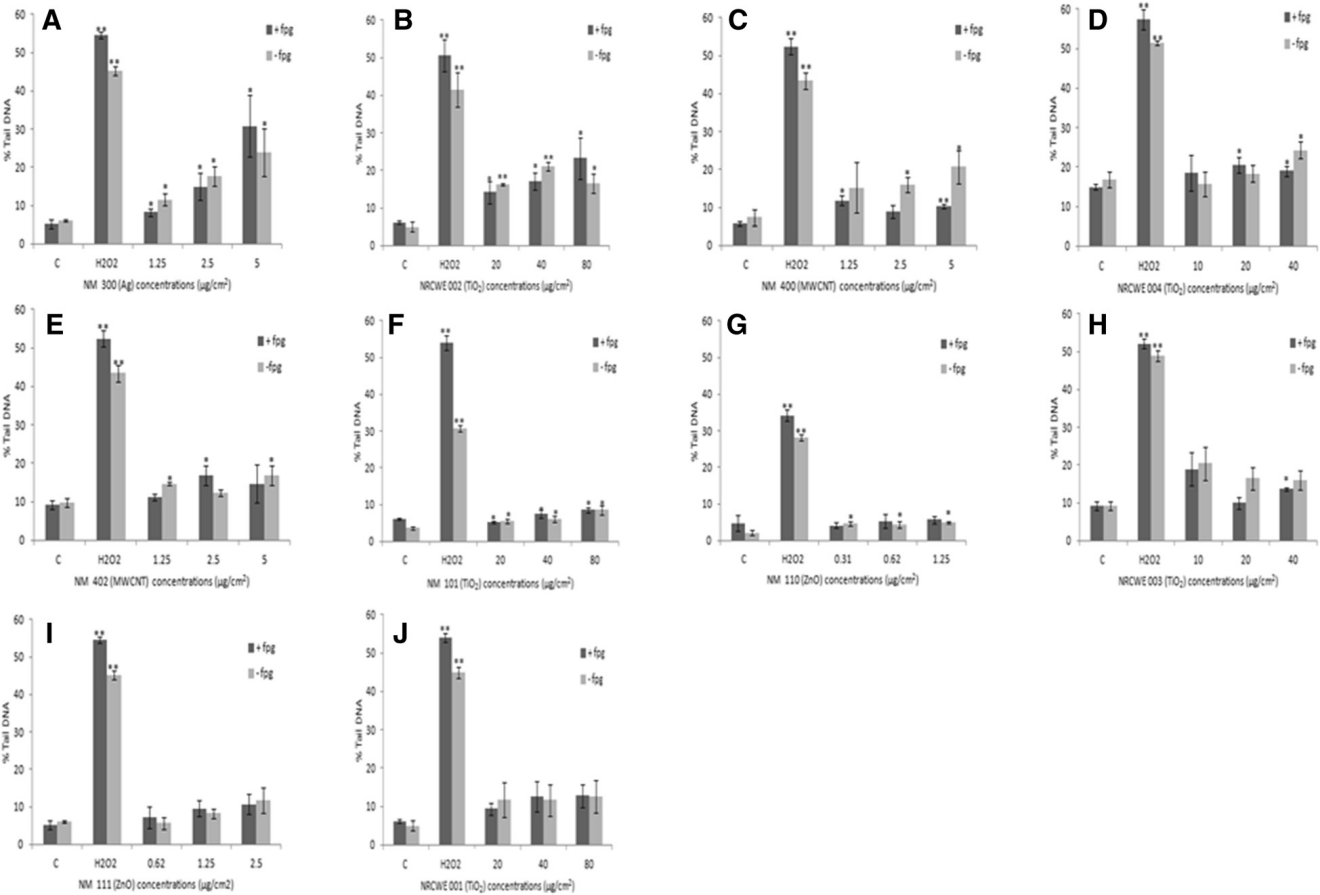
**DNA damage expressed as percent of tail DNA following exposure of the HK-2 cells to the ENPRA panel of engineered nanomaterials.** The cells were exposed to cell medium (control), 60 μM H_2_O_2_ and NMs for 4 hr. Values represent mean ± SEM (n = 3), significance indicated by * = p < 0.05 and ** = p < 0.005, when material treatments are compared to the control. **A**) NM 300 **B**) NRCWE 002 **C**) NM 400 **D**) NRCWE 004 **E**) NM 402 **F**) NM 101 **G**) NM 110 **H**) NRCWE 003 **I**) NM 111 **J**) NRCWE 001. The LC_20_ ± one serial dilution has been used for the majority of NMs (NM 110, NM 111, NM 300, NM 400, NM 402, NRCWE 003 and NRCWE 004). For NMs in which an LC_20_ was not reached the three highest concentrations were utilised.

In order to investigate the possible DNA damage caused by the panel of nanomaterials, HK-2 cells were exposed to the NMs for 4 hr. In this study we chose the LC_20_ value for each individual NM plus one concentration above (2× LC_20_) and one below (0.5× LC_20_). For the TiO_2_ NMs (NM 101, NRCWE 001 and NRCWE 002) were an LC_20_ was not reached the three highest concentrations were utilised. We observed that DNA damage was most evident following exposure to NM 300 (Ag) and NRCWE 002 (positively charged TiO_2_) (Figure 3A, B). We also noted a small but significant increase in the percentage of tail DNA following exposure to five of the other eight NMs investigated (ZnO - NM 111, TiO_2_ - NRCWE 001 and 003 TiO_2_ NMs being the exception) (Figure 3H, I and J).

## Discussion

This study was conducted as part of a large consortium (FP7 project – ENPRA) to investigate the hazards associated with a wide range of nanomaterials on a variety of target systems for risk assessment. For this reason, the wide dose response range was used in order to allow calculation of values such as LC_50_ for comparisons between different materials and cell target types both *in vitro* and *in vivo*. For this particular manuscript kidneys were the target of interest.

To date human kidneys have received relatively little attention in terms of a nanotoxicological studies. Due to high blood supply level and ability to concentrate toxins, this organ could be potentially affected by any nanomaterial that reaches the circulatory system. During active and passive transport of molecules during re-absorption across the nephral proximal tubule, this part of the nephron may be at high risk of potential damage from NMs. It has been shown previously that the proximal renal tubule is more susceptible to gold NM toxicity than the distal tubule [20]. In a recent study it was shown that a high concentration of orally administrated Ag NMs accumulated in kidney tissues at a higher wet mass than any other organ [21]. This observation suggests that kidneys are important organs in clearing the NMs from the mammalian system and at potential risk of being damaged by NM exposure. Hence we have chosen an immortalized proximal tubule epithelial cell line from adult human kidney (HK-2) as a well differentiated cell line representing an alternative to primary kidney cells [22]. This study has focused on the impacts of the investigated panel of nanomaterials on cell cytotoxicity, pro-inflammatory cytokine production, intracellular ROS and DNA damage.

Here we have shown that following acute *in vitro* exposure of the cells to the panel of the NMs, they can be segregated into a low (TiO_2_ and MWCNT) and a high toxicity group (Ag, and ZnO) (Table 1). This complements our previous data for a hepatocyte cell line [14] and primary human hepatocytes [23]. We also observed slightly higher cytotoxicity (not significant) to the cells in RPMI-FCS exposed to the Ag and ZnO NMs (higher amounts of protein within the media compared to the K-SFM) utilised in this study. One theory offering an explanation for this could be that the serum interaction with the NMs might make the materials more bio-available to the cells. It is also possible that the cells might be healthier in the K-SFM compared to RPMI with FCS under the conditions in this study.

We noted that the Ag NM (NM 300) was highly toxic to the HK-2 cells (LC_50_ between 4.5 - 10 μg/cm^2^). To our knowledge no study has investigated the toxicity of Ag nanomaterials and human kidney cells *in vitro* however a number of studies have demonstrated that Ag NMs can be highly toxic in other organs and systems both *in vivo* and *in vitro*[14,24,25]. Oxidative stress is often proposed as a mechanism of toxicity [25,26]. Our HE oxidation data show an increase in intracellular ROS levels following exposure to the NM 300. However a note caution is required as this increase in HE positive cells could be due to cell death. We do not believe this to be the case predominately for two reasons. Firstly, only sub-lethal doses were utilised in these experiments. Secondly, the cells were only exposed to NMs for 4 hr in these experiments with the aim of identifying the mechanisms underlying the cytotoxicity. Furthermore we noted that there was an increase in IL6 and IL8 levels following exposure of the cells to the Ag NMs (highest release at intermediated doses – suggesting cytotoxicity (cell death) inhibits cytokine production at the highest doses), as well as a significant increase in genotoxicity following exposure to sub-lethal concentrations of the nanomaterial. This indicates that Ag NMs are highly genotoxic to human kidney cells. So far a number of other studies have confirmed dose dependent DNA damage after Ag NM treatment to a liver cell line [27] or genotoxicity in testicular cells [28].

Next we have shown that the two ZnO NMs were highly toxic (with the LC_50_ around 0.9 - 2.5 μg/cm^2^) to the renal cells. Our findings are similar to a previous study in which HK-2 cells were shown to be highly susceptible to exposure to ZnO NMs (LC_50_ - 2.4 μg/cm^2^) [29]. The toxicity was associated with increased intracellular ROS production which is very similar to our findings here [29]. Notably, exposure to the two zinc oxide NMs showed only a small increase in percentage of tail damage. It is possible that the 4 hr exposure time for genotoxicity was not sufficient to cause larger effects from the two ZnO NMs in this study. Next, we noted a dose dependant increase in IL6 and IL8 secretion from the cells following NM exposure. It has been shown previously that there is an increase of these pro-inflammatory mediators from the kidney following exposure to various antigens [30,31]. In a similar theme exposure of human embryonic kidney cell line HEK 293 to 100 nm ZnO NMs resulted in up-regulation of IL6 and IL8 genes from the cells *in vitro*[32].

We had previously shown that the two ZnO NMs used in this study are highly soluble (40-50%) while less than 1% of Ag (NM 300) was soluble after 24 hr of incubation in complete medium [14]. Therefore there is a real possibility that the high cytotoxicity of the two ZnO materials are in part due to the release of ions, with this scenario being less likely following exposure to the Ag NMs.

It has been shown previously that in sufficient doses TiO_2_ can cause damage to cells and tissue [14,33,34]. Furthermore other studies have shown that after translocation from the primary site of exposure, the NMs can induce oxidative stress - mediated toxicity in many cell types by producing large amounts of free radicals [12,35,36]. In this study we found all five TiO_2_ were of relatively low cytotoxicity to the HK-2 cells (LC_50_ was not reached in the presence of any of the NMs up to 80 μg/cm^2^) with only small increases of oxidative stress presenting cells following exposure to four of the five TiO_2_ NMs. Our findings are similar to a previous study in which exposure of HK-2 cells to a 12 nm TiO_2_ resulted in low toxicity [29]. Similarly, in a study using Caco2 cells it was found that there was low cytotoxicity following 24 hr exposure to TiO_2_ NMs [35]. In addition the data presented here indicate that relatively high TiO_2_ exposure concentrations can induce production of the pro-inflammatory cytokine IL6 and IL8. In a recent study exposure of mice to 6 nm TiO_2_ NM via an intragastric route promoted the expression of IL2, IL4, IL6, IL8, IL10, IL18, IL1-β, TGF-β and IFN-γ from the kidney [37]. Furthermore exposure of C3A cell line *in vitro* also resulted in IL8 secretion at high TiO_2_ concentrations [14,38].

We show a significant increase in genotoxicity following a 4 hr exposure to two of the five TiO_2_ NMs investigated in this study (NRCWE 002 and NRCWE 004 - most notable following exposure to the positively charged TiO_2_ NMs). One possible explanation for this could be that the positively charged NMs enter cells utilising faster and more effective pathways (fast attachment to cell membrane and ingestion) than their neutral and negative counterparts [39-41]. Overall our findings are similar to a recent study in which exposure of Cos-1 monkey kidney fibroblasts to TiO_2_ NMs resulted in significant DNA damage as measured via the comet assay [42].

We found that the MWCNTs tested were relatively non-toxic to the HK-2 cells at the times and concentrations tested. The toxicity of MWCNTs is widely documented, with adverse effects observed in pulmonary [34], hepatic [14], renal [43], dermal cells [44] as well as monocyte [45] and macrophage cells [46]. It has been shown that exposure of NRK-52E cells (an *in vitro* renal model) to MWCNT resulted in low toxicity (LC_25_ following 24 hr exposure) [47] which is similar to our findings in this study. Exposure of the HK-2 cells to the two MWCNTs in this study resulted in a dose dependant increase in both IL6 and IL8 following a 24 hr exposure. Our findings are similar to a recent study in which exposure of HEK 293 cells to two types of MWCNTs (80 and 150 nm) resulted in increased production of IL8 [48]. The HE oxidation assay showed no intracellular ROS following exposure to either of two MWCNTs. This is contradictory to findings from Reddy and colleagues in which cytotoxicity was associated with oxidative stress [48]. Finally we show that short term (4 hr) exposure of the HK-2 cells to the two carbon nanotubes at sub-lethal concentrations resulted in significant DNA damage. Barillet *et al.* also witnessed small but significant genotoxicity following exposure of NRK-52E cells to 100 nm MWCNT which is similar to our findings in this study [43]. In another study exposure of lung A549 cells for 24 hr to -OH functionalized and pristine MWCNTs resulted in a concentration-dependent increase of direct DNA damage [49]. Similarly A549 cells exposed to MWCNTs for 24 hr resulted in an induction of direct DNA with statistical significance reached at 10 μg/ml [50].

No TNF-α or MCP-1 production was observed following exposure to the ENPRA panel of NMs. It has been shown that under certain disease models that kidney cells produce TNF-α [51] and MCP-1 [52,53]. Here we show that exposure to these particular nanomaterials even at very high (in some cases cytotoxic) doses are not sufficient for MCP-1 or TNF-α secretion from the cells.

Overall this study emphasises the importance of the use of sub-lethal NM concentrations in particular with regards to mechanistic studies - otherwise conclusions about cause and effect and the mechanism of action can be misleading or completely missed. It is very possible that low toxicity nanomaterials may still possess sub-lethal effects of toxicological consequence.

## Conclusions

In conclusion, this *in vitro* renal model demonstrated that ZnO and Ag NMs were consistently more potent with respect to cytotoxicity, cytokine production (IL6 and IL8) and intracellular reactive oxygen species production. In comparison the MWCNT and TiO_2_ nanomaterials investigated revealed relatively lower toxicity. We noted that short term sub-lethal exposure to seven of the ten nanomaterials resulted in DNA damage to the cells (most evident following exposure to the Ag and the positively charged TiO_2_ NMs). Studies conducted by project partners are utilising other target cells such as macrophages, lung epithelial cells, fibroblasts, endothelial cells and hepatocytes. *In vivo* studies are also being conducted for comparison with *in vitro* models. All of this data will be combined into a database to be used in risk assessment.

## Competing interests

The authors declare that they are no competing interests.

## Authors’ contributions

AK, SV, KM and LAA have carried out the experiments within this study. SB, BKG, ABS and VS have all been heavily involved in the preparation and revision of the manuscript. All authors have read and approved the final manuscript.

## Pre-publication history

The pre-publication history for this paper can be accessed here:

http://www.biomedcentral.com/1471-2369/14/96/prepub

## References

[B1] Buzea C Pacheco II Robbie K Nanomaterials and nanoparticles: sources and toxicity Biointerpahses 2007 2 18 67 10.1116/1.2815690 20419892

[B2] Edwards-Jones V The benefits of silver in hygiene, personal care and healthcare Lett Appl Microbiol 2009 49 147 152 10.1111/j.1472-765X.2009.02648.x 19515146

[B3] Tetley TD Health effects of nanomaterials Bionanotechnol 2007 35 527 531 10.1042/BST0350527 17511644

[B4] Hoet PHM Hohlfeld IB Salata O Nanoparticles – known and unknown health risks J Nanobiotechnol 2004 2 12 27 10.1186/1477-3155-2-12 PMC544578 15588280

[B5] Papp T Schiffmann D Weiss D Castronova V Vallyathan V Rahman Q Human health implications of nonmaterial exposure Nanotoxicology 2008 2 9 27 10.1080/17435390701847935

[B6] Maynard AD Nanoparticle safety – A perspective from the United States Issues in Environ Sci Technol 2007 24 118 131

[B7] Semmler-Behnke M Wolfgang KG Lipka J Fertsch S Wenk A Takeneka S Schmid G Brandau W Bio-distribution of 1.4 and 18 nm gold particles in rats Small 2008 12 2108 2111 19031432 10.1002/smll.200800922

[B8] Yeh TK Chen JK Lin CH Yang MH Yang CS Chou FI Peir JJ Wang MY Chang WH Tsai MH Tsai HT Lin PP Kinetics and tissue distributions of neutron-activated zinc oxide nanoparticles and zinc nitrate in mice: effects of size and particulate nature Nanotechnology 2012 8 online access 10.1088/0957-4484/23/8/085102 22293282

[B9] Eisner C Ow H Yang TX Jia ZJ Dimitriadis E Li LL Wang K Briggs J Levine M Schnermann J Espey MG Measurement of plasma volume using fluorescent silica-based nanoparticles J Appl Physiol 2012 112 681 687 10.1152/japplphysiol.01068.2011 22174395 PMC3289433

[B10] Roberts RG Louden JD Goodship THJ An assessment of the methods available to determine nutritional equilibrium in patients with chronic and renal failure Nephrol Dial Transplant 2000 15 1906 1908 10.1093/ndt/15.12.1906 11096128

[B11] L’Azou B Jorly J On D Sellier E Moisan F Fluery-Feith J Cambar J Brochard P Ohayon-Courtes C *In vitro* effects of nanoparticles on renal cells Part Fibre Toxicol 2008 5 22 10.1186/1743-8977-5-22 19099552 PMC2621238

[B12] Wang JJ Sanderson BJ Wang H Cyto- and genotoxicity of ultrafine TiO_2_ particles in cultured human lymphoblastoid cells Mutat Res 2007 628 99 106 10.1016/j.mrgentox.2006.12.003 17223607

[B13] Cartiera MS Johnson KM Rajendran V Caplan MJ The uptake and intracellular fate of PGLA nanoparticles in epithelial cells Biomaterials 2009 30 2790 2798 10.1016/j.biomaterials.2009.01.057 19232712 PMC3195413

[B14] Kermanizadeh A Pojana G Gaiser BK Birkedal R Bilaničová D Wallin H Jensen KA Sellergren B Hutchison GR Marcomini A Stone V *In vitro* assessment of engineered nanomaterials using C3A cells: cytotoxicity, pro-inflammatory cytokines and function markers Nanotoxicology 2012 10.3109/17435390.2011.653416 22263564

[B15] Jacobsen NR Pojano G Wallin H Jensen KA Nanomaterial dispersion protocol for toxicological studies in ENPRA. Internal ENPRA Project Report 2010 The National Research Centre for the Working Environment available on request

[B16] Speit G Schütz P Bonzheim I Trenz K Hoffmann H Sensitivity of the FPG protein towards alkylation damage in the comet assay Toxicol Lett 2004 146 151 158 10.1016/j.toxlet.2003.09.010 14643967

[B17] Wilklund SJ Agurelli E Aspects of design and statistical analysis in the Comet assay Mutagenesis 2003 2003 18 167 175 10.1093/mutage/18.2.167 12621073

[B18] Sidorkina OM Laval J Role of lysine-57 in the catalytic activities of Escherichia coli formamidopyrimidine-DNA glycosylase (Fpg protein) Nucleic Acids Res 1998 26 5351 5357 10.1093/nar/26.23.5351 9826758 PMC148015

[B19] Kalyanaraman B Oxidative chemistry of florescent dyes: implications in the detection of reactive oxygen and nitrogen species Biochem Soc Trans 2011 39 1221 1225 10.1042/BST0391221 21936793

[B20] Abdelhalim MAK Jarrar BM The appearance of renal cells cytoplasm degeneration and nuclear destruction might be an indication of GNPs toxicity Lipids Health Dis 2011 10 147 153 10.1186/1476-511X-10-147 21859444 PMC3175180

[B21] Kim YS Song MY Park JD Song KS Ryu HR Chung YH Chang HK Lee JH Oh KH Kelman BJ Hwang IK Yu IJ Subchronic oral toxicity of silver nanoparticles Part Fibre Toxicol 2010 7 20 31 10.1186/1743-8977-7-20 20691052 PMC2928176

[B22] Ryan MJ Johnson G Kirk J Fuerstenberg SM Zager RA Torok-Storb B HK-2: an immortalized proximal tubule epithelial cell line from normal adult human kidney Kidney Int 1994 45 48 57 10.1038/ki.1994.6 8127021

[B23] Kermanizadeh A Gaiser BK Ward MB Stone V Primary human hepatocytes vs. hepatic cell line – assessing their suitability for *in vitro* nanotoxicology Nanotoxicology 2012 10.3109/17435390.2012.734341 23009365

[B24] Gaiser BK Fernandes TF Jepson MA Lead JR Tylor CR Baalousha M Biswas A Britton GJ Cole PA Johnston BD Ju-Nam Y Rosenkranz P Scown TM Stone V Interspecies comparison on the uptake and toxicity of silver cerium dioxide nanoparticles Environ Toxicol Chem 2012 31 144 154 10.1002/etc.703 22002553

[B25] Kim HR Kim MJ Lee SY Oh SM Chung KH Genotoxic effects of silver nanoparticles stimulated by oxidative stress in human bronchial epithelial (BEAS-2B) cells Mutat Res–Gen Toxicol Environ Mutagen 2011 726 129 135 10.1016/j.mrgentox.2011.08.008 21945414

[B26] Piao MJ Kang KA Lee IK Kim HS Kim S Choi JY Choi J Hyun JW Silver nanoparticles induce oxidative cell damage in human liver cells through inhibition of reduced glutathione and induction of mitochondrial involved apoptosis Toxicol Lett 2011 201 92 100 10.1016/j.toxlet.2010.12.010 21182908

[B27] Kermanizadeh A Gaiser BK Hutchison GR Stone V An *in vitro* liver model – accessing oxidative stress and genotoxicity following exposure of hepatocytes to a panel of engineered nanoparticles Part Fibre Toxicol 2012 9 28 10.1186/1743-8977-9-28 22812506 PMC3546021

[B28] Asare N Instanes C Sandberg WJ Refsnes M Schwarze P Kruszewski M Brunborg G Cytotoxic and genotoxic effects of silver nanoparticles in testicular cells Toxicology 2012 291 65 72 10.1016/j.tox.2011.10.022 22085606

[B29] Pujalté I Passagne I Brouillaud B Tréguer M Durand E Ohayon-Courtès C L'Azou B Cytotoxicity and oxidative stress induced by different metallic nanoparticles on human kidney cells Part Fibre Toxicol 2011 8 10 26 10.1186/1743-8977-8-10 21371295 PMC3058043

[B30] Dudas PL Sague SL Elloso MM Farrell FX Proinflammtory/Profibiotc effects of interlukin 17A on human proximal tubule epithelium Nephron Exp Nephrol 2011 117 114 123 10.1159/000320177 20924205

[B31] Shing CM Adams MJ Fassett RG Coombes JS Nutritional compounds influence tissue factor expression and inflammation of chronic kidney patients *in vitro* Nutrition 2011 27 967 972 10.1016/j.nut.2010.10.014 21295946

[B32] Dua P Chaudhari KN Lee CH Chaudhari NK Hong SW Yu JS Kim S Lee DK Evaluation of toxicity and gene expression changes triggered by oxide nanoparticles Bull Korean Chem Soc 2011 32 2051 2057 10.5012/bkcs.2011.32.6.2051

[B33] Hu RP Zheng L Zhang T Gao GD Cui YL Cheng Z Cheng J Hong MM Tang M Hong FS Molecular mechanism of hippocampal apoptosis of mice following exposure to titanium dioxide nanoparticles J Hazard Mater 2011 191 32 40 10.1016/j.jhazmat.2011.04.027 21570177

[B34] Ji Z Zhang D Li L Shen X Deng X Dong L Wu M Liu Y The hepatotoxicity of multi-walled carbon nanotubes in mice Nanotechnology 2009 20 445101 10.1088/0957-4484/20/44/445101 19801780

[B35] Jin CY Zhu BS Wang XF Lu QH Cytotoxicity of Titanium dioxide nanoparticles in mouse fibroblast cells Chem Res Toxicol 2008 21 1871 1877 10.1021/tx800179f 18680314

[B36] Kang SJ Kim BM Lee YJ Chung HW Titanium dioxide nanoparticles trigger p53-mediated damage response in peripheral blood lymphocytes Environ Mol Mutagen 2008 49 399 405 10.1002/em.20399 18418868

[B37] Gui S Zhang Z Zheng L Cui Y Liu X Li N Sang X Sun Q Gao G Cheng Z Wang L Tang M Hong F Molecular mechanism of kidney injury of mice caused by exposure to titanium dioxide nanoparticles J Hazard Mater 2011 195 365 370 21907489 10.1016/j.jhazmat.2011.08.055

[B38] Monteiller C Tran L MacNee W Faux S Jones A Miller B Donaldson K The pro-inflammatory effects of low toxicity low solubility particles, nanoparticles and fine particles, on epithelial cells *in vitro:* the role of surface area Occup Environ Med 2007 64 609 615 10.1136/oem.2005.024802 17409182 PMC2092561

[B39] Arvizo RR Miranda OR Thompson MA Pabelick CM Bhattacharya R Robertson JD Rotello VM Prakash YS Mukherjee P Effects of nanoparticle surface charge at the plasma membrane and beyond Nano Lett 2010 10 2543 2548 10.1021/nl101140t 20533851 PMC2925219

[B40] Schweiger C Hartmann R Zhang F Parak WJ Kissel TH Gil PR Quantification of the internalization patterns of superparamagnetic iron oxide nanoparticles with opposite charge J Nanobiotechnology 2012 10 28 10.1186/1477-3155-10-28 22781560 PMC3431280

[B41] Verma A Stellacci F Effects of surface properties on nanoparticle-cell interactions Small 2009 6 12 21 19844908 10.1002/smll.200901158

[B42] Magdolenova Z Bilanicova D Pojana G Fjellsbo LM Hudecova A Hasplove K Marcomini A Dusinska M Impact of agglomeration and different dispersions of titanium dioxide nanoparticles on the human related *in vitro* cytotoxicity and genotoxicity J Environ Monit 2012 14 455 464 10.1039/c2em10746e 22277962

[B43] Barillet S Simon-Deckers A Herlin-Boime N Mayne-L’Hermite M Reynaud C Cassio D Gouget B Carriere M Toxicological consequences of TiO_2_, SiC nanoparticles and multi-walled carbon nanotubes exposure in several mammalian cell types: an *in vitro* study J Nanopart Res 2010 12 61 73 10.1007/s11051-009-9694-y

[B44] Kishore AS Surekha P Murthy PB Assessment of the dermal and ocular irritation potential of multi-walled carbon nanotubes by using *in vitro* and *in vivo* methods Toxicol Lett 2009 191 268 274 10.1016/j.toxlet.2009.09.007 19770026

[B45] De Nicola M Nuccitelli S Gattia DM Traversa E Magrini A Bergamaschi A Ghibelli L Effects of carbon nanotubes on human monocytes. Natural compounds and their role in apoptotic cell signalling pathways Ann N Y Acad Sci 2009 1171 600 605 10.1111/j.1749-6632.2009.04892.x 19723110

[B46] Hirano S Kanno S Furuyama A Multi-walled carbon nanotubes injure the plasma membrane of macrophages Toxicol Appl Pharmacol 2008 232 244 251 10.1016/j.taap.2008.06.016 18655803

[B47] Simon A Reynaud C Mayne M Herlin N Desqueyroux H Gouget B Carriere M Cytotoxicity of metal oxide nanoparticles and multiwalled carbon nanotubes to lung, kidney and liver cells Met Ions Biol Med 2008 10 310 314

[B48] Reddy ARN Reddy YN Krishna DR Himabindu V Multi walled carbon nanotubes induce oxidative stress and cytotoxicity in human embryonic kidney (HEK293) cells Toxicology 2010 272 11 16 10.1016/j.tox.2010.03.017 20371264

[B49] Ursini CL Cavallo D Fresegna AM Ciervo A Maiello R Buresti G Casciardi S Tombolini F Bellucci S Iavicolli S Comparative cyto-genotoxicity assessment of functionalized and pristine multi-walled carbon nanotubes on human lung epithelial cells Toxicol In Vitro 2012 26 831 840 10.1016/j.tiv.2012.05.001 22640919

[B50] Cavallo D Fanizza C Ursini CL Casciardi S Paba E Ciervo A Fresegna AM Buresti G Tombolini F Bellucci S Iavicolli S Multi-walled carbon nanotubes induce cytotoxicity and genotoxicity in human lung epithelial cells J Appl Toxicol 2012 32 454 464 10.1002/jat.2711 22271384

[B51] Zuo N Suzuki Y Sugaya T Osaki K Kanaguchi Y Wang LN Tomino Y Protective effects of tubular liver type fatty acid-binding protein against glomerular damage in murine IgA nephropathy Nephrol Dial Transplant 2011 26 2127 2137 10.1093/ndt/gfq687 21109611

[B52] Wittlinger M Schlapfer M De Conno E Zgraggen BR Reyes L Booy C Schimmer RC Seifert B Burmeister MA Spahn DR Beck-Schimmer B The effect of hydroxyethyl starches (HES 130/0.42 and HES 200/0.5) on activated renal tubular epithelial cells Anesth Analg 2010 110 531 540 10.1213/ANE.0b013e3181c03c97 19910630

[B53] Zehnder D Quinkler M Eardley KS Bland R Lepenies J Hughes SV Raymond NT Howie AJ Cockwell P Stewart PM Hewiston M Reduction of the vitamin D hormonal system in kidney disease is associated with increased renal inflammation Kidney Int 2008 74 1343 1353 10.1038/ki.2008.453 18784644 PMC2737358

